# Metacognitive therapy home-based self-help for cardiac rehabilitation patients experiencing anxiety and depressive symptoms: study protocol for a feasibility randomised controlled trial (PATHWAY Home-MCT)

**DOI:** 10.1186/s13063-018-2826-x

**Published:** 2018-08-16

**Authors:** Adrian Wells, Kirsten McNicol, David Reeves, Peter Salmon, Linda Davies, Anthony Heagerty, Patrick Doherty, Rebecca McPhillips, Rebecca Anderson, Cintia Faija, Lora Capobianco, Helen Morley, Hannah Gaffney, Calvin Heal, Gemma Shields, Peter Fisher

**Affiliations:** 1 The University of Manchester, School of Psychological Sciences, Faculty of Biology, Medicine and Health, Rawnsley Building, Manchester Royal Infirmary, Oxford Road, Manchester, M13 9WL UK; 2 Greater Manchester Mental Health NHS Foundation Trust, Rawnsley Building, Manchester Royal Infirmary, Oxford Road, Manchester, M13 9WL UK; 3 The University of Manchester, NIHR School for Primary Care Research, Manchester Academic Health Science Centre, Williamson Building, Oxford Road, Manchester, M13 9PL UK; 4 University of Liverpool, Institute of Psychology, Health and Society, Waterhouse Building, Block B, Brownlow Street, Liverpool, L69 3GL UK; 5 The Royal Liverpool and Broadgreen University Hospitals NHS Trust, Prescot Street, Liverpool, L7 8XP UK; 6 Division of Population Health, Health Services Research and Primary Care, School of Health Sciences, Faculty of Biology, Medicine and Health, The University of Manchester, Centre for Health Economics, Jean McFarlane Building, Oxford Road, Manchester, M13 9PL UK; 7 The University of Manchester, School of Medical Sciences, Core Technology Facility, Grafton Street, Manchester, M13 9NT UK; 8 Central Manchester Foundation Trust, Manchester Royal Infirmary, Oxford Road, Manchester, M13 9WL UK; 9 Department of Health Sciences, University of York, Seebohm Rowntree Building, York, YO10 5DD UK; 10 Division of Neuroscience and Experimental Psychology, The University of Manchester, School of Biological Sciences, Oxford Road, Manchester, M13 9PL UK; 11 Division of Psychology and Mental Health, The University of Manchester, School of Health Sciences, Oxford Road, Manchester, M13 9PL UK; 12 The University of Manchester, Centre for Biostatistics, Faculty of Biology, Medicine and Health, Manchester Academic Health Science Centre, Manchester, M13 9PL UK

**Keywords:** Metacognitive therapy, Rumination, Worry, Anxiety, Depression, Cardiac rehabilitation, Self-help, Home therapy, Psychological intervention, Heart disease

## Abstract

**Background:**

Anxiety and depression are common among patients attending cardiac rehabilitation services. Currently available pharmacological and psychological interventions have limited effectiveness in this population. There are presently no psychological interventions for anxiety and depression integrated into cardiac rehabilitation services despite emphasis in key UK National Health Service policy. A new treatment, metacognitive therapy, is highly effective at reducing anxiety and depression in mental health settings. The principal aims of the current study are (1) to evaluate the acceptability of delivering metacognitive therapy in a home-based self-help format (Home-MCT) to cardiac rehabilitation patients experiencing anxiety and depressive symptoms and conduct a feasibility trial of Home-MCT plus usual cardiac rehabilitation compared to usual cardiac rehabilitation; and (2) to inform the design and sample size for a full-scale trial.

**Methods:**

The PATHWAY Home-MCT trial is a single-blind feasibility randomised controlled trial comparing usual cardiac rehabilitation (control) versus usual cardiac rehabilitation plus home-based self-help metacognitive therapy (intervention). Economic and qualitative evaluations will be embedded within the trial. Participants will be assessed at baseline and followed-up at 4 and 12 months. Patients who have been referred to cardiac rehabilitation programmes and have a score of ≥ 8 on the anxiety and/or depression subscales of the Hospital Anxiety and Depression Scale will be invited to take part in the study and written informed consent will be obtained. Participants will be recruited from the National Health Service in the UK. A minimum of 108 participants will be randomised to the intervention and control arms in a 1:1 ratio.

**Discussion:**

The Home-MCT feasibility randomised controlled trial will provide evidence on the acceptability of delivering metacognitive therapy in a home-based self-help format for cardiac rehabilitation patients experiencing symptoms of anxiety and/or depression and on the feasibility and design of a full-scale trial. In addition, it will provide provisional point estimates, with appropriately wide measures of uncertainty, relating to the effectiveness and cost-effectiveness of the intervention.

**Trial registration:**

ClinicalTrials.gov, NCT03129282, Submitted to Registry: 11 April 2017.

**Electronic supplementary material:**

The online version of this article (10.1186/s13063-018-2826-x) contains supplementary material, which is available to authorized users.

## Background

There is strong evidence demonstrating that anxiety and depressive symptoms are common among heart disease patients [1–5]. In addition, the European Association of Preventive Cardiology has emphasised that symptoms of anxiety and depression in heart disease patients play a key role in the success of cardiovascular rehabilitation (CR) programmes [6]. Anxiety and depression are associated with lower rates of adherence to treatment, a higher prevalence of high-risk behaviours (e.g. smoking), and increased risk of further cardiac events and mortality [3, 7–11]. Specifically, a meta-analysis found that depression is a risk factor for mortality in heart disease patients [12], and anxiety and depressive symptoms predicted mortality in percutaneous coronary intervention patients at a 10-year follow-up [13]. Furthermore, two recent studies in this population found that anxiety and depression predicted future symptoms of emotional distress, higher rates of hospital readmissions, higher costs to the healthcare system, lower quality of life, and poorer prognosis [14, 15]. Consequently, it is crucial to identify and treat symptoms of anxiety and depression effectively to ensure that CR programmes have better clinical outcomes, improve the quality of life of heart disease patients, and reduce health service costs.

In 2010, the Department of Health implemented the CR commissioning pack to improve cost-effectiveness in CR services [16], and more recently the European Association for Preventive Cardiology issued details of the core components and standards for secondary prevention in the clinical management of patients with cardiovascular diseases [6]. Both include comprehensive service specifications where psychological assessment and support are advocated throughout [6, 16], specifically assessment of patients’ psychological needs, including anxiety and depression, and interventions targeting them, using evidence-based approaches. Both organisations further advocate that such comprehensive CR programmes should be delivered by appropriately trained professionals [6, 16]. CR programmes aim to facilitate recovery after a heart event, promote healthy behaviours, improve lifestyle risk factors, reduce the risk of further related problems, and improve patients’ emotional wellbeing (e.g. anxiety, depression) and health-related quality of life. CR programmes are usually delivered as centre-based group programmes in hospital or community settings [16]. In a survey of CR services in 28 European countries, two thirds of countries reported a 30% or lower provision of an outpatient (Phase II) CR programme [17]. In the UK, 49% of referred patients decline participation [18], with lower uptake and adherence in certain groups including people experiencing emotional distress [19]. Home-based programmes have been introduced to widen access and participation in CR. Two home-based self-help packages have been successfully integrated into CR programmes into the National Health Service (NHS) [20], the Heart Manual [21] and the Angina Plan (www.anginaplan.org.uk) [22]. These programmes include a manual that patients complete at home and are facilitated by trained healthcare professionals either face-to-face or by telephone. Both home-based approaches follow the CR commissioning pack guidelines and comprise a number of components, including education about heart disease, medication and dietary recommendations, an exercise/activity plan, lifestyle change advice, information and strategies to manage anxiety and depression, and a relaxation CD. These two home-based self-help packages are utilised by over 20,000 heart disease patients annually in the UK, and there is an increasing trend towards self-help approaches [23–25]. The Department of Health CR Commissioning Pack [16] and recent British Association for Cardiovascular Prevention and Rehabilitation guidelines [23] recommend that the choice of home-based CR as an alternative to centre-based programmes should be provided to all patients as part of a menu-based approach.

Randomised controlled trials showed no differences between home-based and centre-based programmes concerning mortality, cardiac events, exercise capacity, smoking cessation, blood pressure, total cholesterol, anxiety and depression, and health-related quality of life [20, 26–28]. However, adherence to treatment in home-based programmes was superior [26].

Recent UK CR data [29] demonstrated that 28% of patients experience symptoms of anxiety within the clinical range at the start of CR and only 6% move into the normal anxiety range after CR; however, the variation of improvement at the local level ranges from − 9% (some patients get worse) to 28%. A further 19% of patients report symptoms of depression at the beginning of CR and only 6% move into the normal depression category following CR, with the variation of improvement at the local level ranging from − 8% to 24%. Thus, most patients continue to experience anxiety and depression symptoms within the clinical range after completing CR programmes. Consequently, it is imperative that effective interventions for anxiety and depression are integrated into both centre-based and home-based CR programmes.

There is a need to improve the treatment of anxiety and depression in CR. In contrast, treatment in mental health settings has been improving. There is strong evidence supporting the effectiveness of metacognitive therapy (MCT) [30], a new psychological therapy that could offer a potentially effective intervention for treating depression and anxiety in patients attending CR programmes. MCT is founded on an evidenced-based model called the Self-Regulatory Executive Function model [31, 32], which proposes that emotional distress is maintained by a style of thinking called the cognitive attentional syndrome (CAS). The CAS is characterised by repetitive negative thinking (worry and/or rumination), focusing attention on threat (e.g. thoughts, physical symptoms, emotions), and maladaptive coping strategies (e.g. cognitive avoidance, behavioural avoidance, alcohol/substance misuse). This thinking style is driven by underlying metacognitive beliefs, which lead to prolonged negative processing and maintenance of psychological distress. Metacognitive beliefs can be divided into positive beliefs that focus on the usefulness of worry, rumination, threat monitoring and other coping strategies (e.g. avoidance), for example, “Worrying helps me to avoid problems in the future”. Negative metacognitive beliefs, on the other hand, focus on the uncontrollability, danger, importance and meaning of thoughts, such as “My worrying thoughts persist, no matter how I try to stop them”. In the Self-Regulatory Executive Function model, the CAS and metacognitive beliefs are transdiagnostic, meaning that they maintain all forms of emotional disorder. There is evidence supporting the role of these factors in emotional distress across a wide range of health conditions including cancer [33], Parkinson’s disease [34, 35] and chronic fatigue syndrome [36, 37]. In addition, a recent systematic review demonstrated that unhelpful styles of thinking (worry and/or rumination) predicted depression, anxiety and emotional distress in people with a range of long-term health conditions including heart disease [38].

Randomised controlled trials have shown that MCT is an effective psychological treatment for anxiety and depression [39–41]. In addition, MCT has been identified as a high intensity psychological intervention for treating individuals with a diagnosis of generalised anxiety disorder by the National Institute for Health and Care Excellence [42]. In a recent meta-analysis of the efficacy of MCT for anxiety and depression it was found to be more effective than waitlist control groups and cognitive behavioural therapy [43].

In summary, MCT offers an effective psychological approach to treating anxiety and depression in mental health settings and there is preliminary evidence of MCT effectiveness across a range of medical conditions (i.e. cancer, Parkinson’s disease and chronic fatigue syndrome). As MCT is based on the transdiagnostic processes underlying psychological distress, it could be that it has the potential to generalise well to heart disease patients attending CR programmes who suffer from anxiety and depression. Therefore, the PATHWAY programme aims to provide evidence of MCT integrated into CR services investigating MCT in two formats as a centre-based group intervention (Group-MCT) and a home-based self-help intervention (Home-MCT). The Group-MCT intervention has been described in Wells et al. [44] and the Home-MCT intervention is described in the present article. Such treatment options are in line with a modern era menu-based approach and could add to the delivery of CR programmes by increasing the range of options for accessing psychological support that do not require referral to mental health services.

### Aims

There is currently no evidence on MCT delivered in a home-based self-help format among patients referred to CR services. Therefore, the aims of the PATHWAY Home-MCT trial are to (1) evaluate the acceptability of the Home-MCT intervention to CR patients and conduct a feasibility trial of Home-MCT plus usual CR compared to usual CR; (2) collect data on patient variables and outcome measures (e.g. emotional distress, post-traumatic stress, metacognitive beliefs, quality of life) to inform the design and sample size for a full-scale trial; (3) establish provisional evidence of the effectiveness of Home-MCT at reducing anxiety and depression symptoms; (4) obtain qualitative data to help refine the presentation and delivery of Home-MCT for a full-scale trial; and (5) provide preliminary estimates of the cost-effectiveness of Home-MCT.

## Methods

### Design

**Fig. 1:**
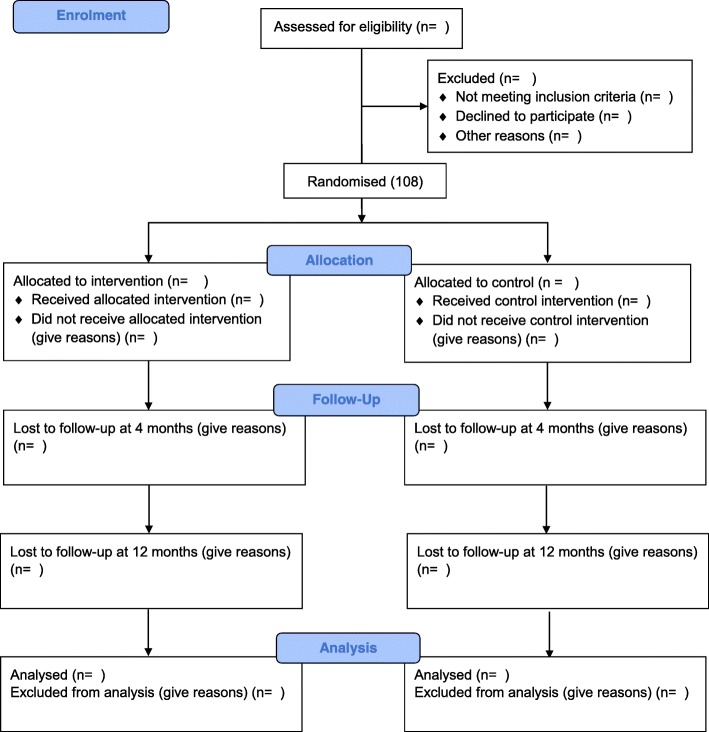
CONSORT 2010 Standard Randomised Control Trials flow diagram (numbers are target values)

**Fig. 2:**
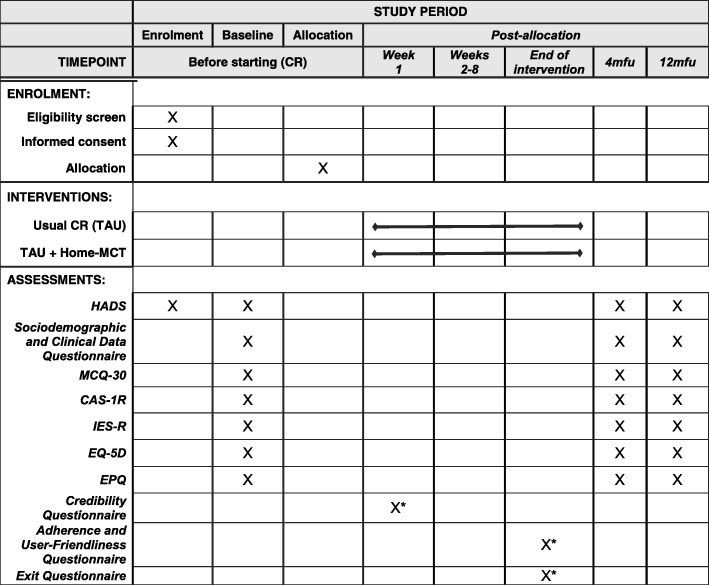
Schedule of enrolment, interventions and assessments. *CR* cardiac rehabilitation, *TAU* treatment as usual, *Home-MCT* home-based metacognitive therapy, *HADS* Hospital Anxiety and Depression Scale, *MCQ-30* Metacognitions Questionnaire 30, *CAS-1R* Cognitive Attentional Syndrome Scale-1 Revised, *IES-R* Impact of Event Scale-Revised, *EQ-5D* European Quality of Life 5 Dimensions, *EPQ* Economic Patient Questionnaire. *Intervention participants only

The PATHWAY Home-MCT is a single-blind feasibility randomised controlled trial with 4- and 12-month follow-up comparing Home-MCT alongside usual CR (intervention) versus usual CR (control). Qualitative and economic evaluations will be embedded within the trial. Figure 1 shows an overview of the trial design according to the CONSORT guidelines [45]. The Recommendations for Interventional Trials 2013 (SPIRIT) Checklist [46, 47] is included in Additional file 1. Figure 2 shows a schedule of enrolment, interventions and assessments.

### Study setting

This feasibility trial is part of a 5-year programme of research funded by the National Institute for Health Research and sponsored by Greater Manchester Mental Health NHS Foundation Trust (GMMH). The study will take place in England, at NHS sites offering CR.

### Trial population

The trial population are heart disease patients referred to CR programmes at two NHS sites in England, the Aintree University Hospitals NHS Foundation Trust and Bolton NHS Foundation Trust.

### Eligibility criteria

Patients will be invited to take part in the trial if they meet the following inclusion criteria:

1. Meets Department of Health and/or British Association for Cardiovascular Prevention and Rehabilitation CR eligibility criteria. Thus, the patient will have at least one of the following: acute coronary syndrome, revascularisation, stable heart failure, stable angina, implantation of cardioverter defibrillators/cardiac resynchronisation devices, heart valve repair/replacement, heart transplantation and ventricular assist devices, adult congenital heart disease, other atypical heart presentation.
2. A score of ≥ 8 on the anxiety and/or depression subscales of the Hospital Anxiety and Depression Scale (screening HADS) [48].
3. Minimum of 18 years old.
4. A competent level of English language skills (able to read, understand and complete questionnaires in English).

Participants will be excluded if they meet any of the following criteria:

1. Cognitive impairment which precludes informed consent or ability to participate.
2. Acute suicidality.
3. Active psychotic disorders (i.e. two (or more) of the following: delusions, hallucinations, disorganised speech, grossly disorganised or catatonic behaviour, negative symptoms).
4. Current drug/alcohol abuse (a maladaptive pattern of drinking, leading to clinically significant impairment or distress).
5. Concurrent psychological intervention for emotional distress that is not part of usual care.
6. Antidepressant or anxiolytic medications initiated in the previous 8 weeks.
7. Life expectancy of less than 12 months.

Patients who are ineligible or decline to participate will continue to receive care along the usual CR pathway.

### Recruitment and allocation

Patients referred to the CR programme at each site will complete the HADS [48] as part of their routine assessments. If a patient scores 8 or above on the anxiety and/or the depression subscale of the screening HADS, CR nurses will screen the patient’s medical notes to check for eligibility. Patients deemed eligible for the trial will be provided with information about the study (i.e. invitation flyer and participant information sheet) and CR staff will seek an expression of interest either face-to-face or by telephone.

Interested eligible patients will be contacted by a research assistant, who will arrange to meet with them prior to their first CR session at a convenient location (i.e. NHS Trusts or patients’ home). A research assistant or person designated by the principal investigator at each hospital site will take written informed consent. Baseline questionnaires will be collected face-to-face or over the phone and will be administered prior to the participant’s first CR session. The completion of the questionnaires will take between 35 and 45 minutes. After consent and baseline assessments, participants will be randomised via telephone/email link to the Centre for Biostatistics at the University of Manchester. A randomisation sequence will be created using Stata 14 statistical software [49], stratified by sex, site and screening HADS scores [48]. Participants will be allocated to trial arms in a 1:1 allocation using randomised block sizes of 4 and 6. Research assistants who are not blind to treatment allocation will inform each participant of their group allocation. Research assistants who are collecting assessment data (i.e. baseline, 4 and 12 months post-randomisation) will be blind to treatment allocation, as will the chief investigator (AW) and the trial statistician (DR).

### Trial conditions

#### Usual CR (control group)

Participants in the control group will be invited to join the usual CR programme offered at their site. The majority of patients at both sites will attend group-based programmes that include exercise and educational sessions. The exercise sessions will include warm up exercises, a circuit of cardiovascular exercises at the appropriate intensity for each patient, cool down and stretching exercises. The educational sessions will cover risk factors that contribute to heart problems, signs, symptoms and medication, the importance of eating healthily and exercising, and a one-off talk about stress (e.g. signs, symptoms and relaxation techniques). Participants will be offered weekly sessions over a period of 6–8 weeks and each session will last approximately 2 hours. A small number of patients at each site will receive home-based CR, which comprises similar components to the group programme tailored to participants’ individual needs. The study sites will offer additional support to participants experiencing anxiety and/or depressive symptoms based on CR staff’s clinical judgement of need; Aintree University Hospitals NHS Foundation Trust will offer a 1-hour occupational therapy appointment and Bolton NHS Foundation Trust will offer individual counselling. Furthermore, patients who are found to have particularly high levels of depression or anxiety will be referred to their GP.

#### Home-MCT alongside usual CR (intervention group)

The intervention group will receive Home-MCT in addition to the usual CR.

Home-MCT consists of a self-help manual comprising six modules. Members of the PATHWAY programme’s service user advisory group provided feedback on the structure and user-friendliness of the Home-MCT manual. Suggested changes were implemented before commencing recruitment to the trial.

Participants will complete the Home-MCT manual at their own pace over 6–8 weeks. It is anticipated that participants will complete one module per week, but flexibility in rate of progress will be permitted. The Home-MCT modules focus on modifying the specific metacognitive beliefs and processes that maintain anxiety and depression. Modules comprise well-specified techniques for developing new strategies to overcome worry and rumination, and modifying the metacognitive beliefs that maintain unhelpful patterns of thinking. Participants will be instructed to practise the techniques introduced in each module and to complete tasks between modules.

Participants will receive three scripted telephone calls (up to 30 minutes each) from trained CR staff (e.g. nurses, occupational therapists) over the course of the intervention. The first telephone call will provide an overview of the Home-MCT manual and the remaining two will offer support and guidance on completing the modules and implementing Home-MCT strategies. The first telephone call will be delivered before the participant starts the manual and the remaining ones after the completion of modules 2 and 4. The CR staff will be asked to follow a structured script when making telephone contact. Their role is to provide support and guidance on completing Home-MCT.

#### CR staff training and supervision for the implementation of scripted telephone calls

At least two members of each CR team will be specifically trained to deliver the telephone support calls. Training will consist of two full-day workshops to enable the CR practitioners to become familiar with the implementation of, and to role-play, the telephone scripts. CR staff will have basic knowledge of the metacognitive model; however, they will not be skilled in the delivery of MCT and will not be required to deliver MCT.

Telephone calls provided by CR staff will be audio-recorded and transcribed if a participant has given their written informed consent to ensure consistency of delivery of the support across sites and to examine adherence to the telephone script.

### Data collection: Participant timeline

Participants will complete assessments at three time-points, namely baseline (pre-CR), 4-months post-randomisation and 12-months post-randomisation (Fig. 1).

To minimise attrition rates, participants will be offered a number of options for completing follow-up measures, including postal return, online, by telephone or face-to-face (at NHS sites or participants’ homes). The participant’s time involvement in the study is 12 months. Participants will be compensated for completing each assessment, receiving a £5 voucher at baseline and a £10 voucher at the 4- and 12-month follow-ups.

#### Criteria for discontinuation of participants

Participants may withdraw from the study at any time without giving a reason and without any consequences to themselves, their healthcare or their ability to take part in future research. Participants who withdraw will continue to receive usual care. Participants can also be withdrawn at the request of the Chief Investigator, but this would only happen if a participant’s life or long-term health or welfare is at risk from continued participation in the study.

### Outcomes

The principal outcomes are acceptability and feasibility. Acceptability of the Home-MCT intervention will be assessed primarily using a set of three participant self-report questionnaires relating to credibility, user-friendliness and adherence to the intervention, as described below. The feasibility of conducting a full trial will be evaluated principally in terms of the ability to recruit and retain participants and to collect high quality complete data on the required measures across the timeline of the study.

### Measures

#### Credibility questionnaire

The credibility of the intervention has been defined as (1) how logical Home-MCT seems to participants, (2) how successful Home-MCT seems in reducing levels of emotional distress, and (3) how confident the participants feel in recommending this intervention to someone experiencing similar problems. The Credibility Questionnaire includes three questions assessing these dimensions, with each rated using a scale from 0 to 100. The measure has been adapted from the Credibility/Expectancy Questionnaire developed by Devilly and Borkovec [50]. The Credibility Questionnaire aims to assess how believable, logical and compelling the intervention is to the participant, rather than post-intervention satisfaction or effectiveness. For this purpose, this questionnaire will be completed after reading the introduction section of the Home-MCT manual and before starting module 1.

#### Adherence and User-Friendliness Questionnaire

The Adherence and User-Friendliness Questionnaire was developed for this study and includes six questions that assess how many modules were completed (ranging from 0 to 6), how accessible, easy to follow, easy to understand and easy to use the Home-MCT manual is, and how much the participant needed the supportive telephone calls. All questions, except for the number of modules completed, are rated on a scale from 0 to 100. This questionnaire will be completed at the end of the intervention, with the time-frame for returning this questionnaire being up to 4-months post-randomisation.

#### Exit Questionnaire

The Exit Questionnaire includes two questions to collect specific details about the intervention, namely (1) “Which modules from the Home-MCT manual have you completed?” and (2) “How much time have you spent to complete each module?” This questionnaire will be completed at the end of the intervention, with the time-frame for returning this questionnaire being up to 4-months post-randomisation.

The following self-report questionnaires will also be completed by all participants at each assessment point (i.e. baseline, 4 months follow-up and 12 months follow-up) to assess symptoms, psychological mechanisms and healthcare use.

#### Hospital anxiety and depression scale (HADS) [48]

The HADS is a 14-item self-report scale assessing anxiety (7 items) and depression (7 items). Respondents rate their emotional distress based on the past 7 days using a 4-point scale (from 0 to 3). Possible scores for each sub-scale range from 0 to 21. Scores from 0 to 7 are categorised as normal, from 8 to 10 mild, from 11 to 14 moderate, and from 15 to 21 severe [47]. The HADS is routinely used in CR [28], and has shown good internal consistency for both subscales (i.e. Cronbach’s alpha, anxiety = 0.85, depression = 0.80, total scale = 0.89) [51].

#### Impact of Events Scale – Revised (IES-R) [52]

The IES-R is a 22-item self-report measure that assesses subjective distress caused by traumatic events. For this study, participants will be instructed to answer the items with respect to their recent heart event. Respondents rate the items based on the past 7 days using a 5-point scale ranging from 0 (not at all) to 4 (extremely). This scale yields a total score ranging from 0 to 88 and three subscale scores: Intrusions, Avoidance and Hyperarousal. Scores of 24 to 32 indicate post-traumatic stress disorder is a clinical concern, whereas scores of 33 to 88 indicate a diagnosis of post-traumatic stress disorder [52]. The IES-R subscales have good internal consistency (Cronbach’s alphas range from 0.79 to 0.91) [52].

#### Metacognitions Questionnaire 30 (MCQ-30) [53]

The MCQ-30 is a 30-item self-report scale that measures five different domains of metacognition (cognitive confidence, positive beliefs about worry, cognitive self-consciousness, negative beliefs about uncontrollability and danger, need to control thoughts). Respondents rate how much they “generally agree or disagree” with the statements presented on a 4-point scale (1, do not agree; 2, agree slightly; 3, agree moderately; 4, agree very much). This questionnaire yields a total score ranging from 30 to 120 and four subscale scores. High scores indicate, respectively, lack of cognitive confidence, more positive or negative beliefs about worry, increased tendency towards cognitive self-consciousness, and greater belief in the need to control thoughts. The MCQ-30 subscales have good internal consistency (Cronbach’s alphas range from 0.72 to 0.93) [53–55].

#### European Quality of Life 5 Dimensions 5 Levels (EQ-5D-5 L) [56, 57]

The ED-5D-5 L is a standardised questionnaire for use as a measure of health status and its use is recommended in the National Institute for Health and Clinical Excellence guidelines for economic evaluation [58]. The tool assesses five health dimensions: mobility, self-care, usual activities, pain/discomfort and anxiety/depression. Each dimension has five response options: no problems, slight problems, moderate problems, severe problems and extreme problems. In addition, respondents also rate their overall health on a 0–100 vertical visual analogue scale. The measure has been shown to be valid and reliable in a cardiovascular population [59].

#### Economic Patient Questionnaire (EPQ)

The EPQ is designed to collect data on outpatient services and non-hospital-based health and social care use. The EPQ assesses three areas, namely use of primary and community-based health services, social support services accessed outside the hospital, and aids and equipment used as part of care.

#### Cognitive Attentional Syndrome Scale-1 revised (CAS-1R)

The CAS-1R has been adapted for the present study from the original Cognitive Attentional Syndrome Scale-1 [30], a 16-item self-report questionnaire assessing an individual’s metacognitive beliefs, knowledge and strategies. The CAS-1R includes 10-items assessing the degree to which individuals have been worrying and/or focusing attention on threats, the degree to which individuals hold negative metacognitive beliefs about worry and the degree to which individuals hold positive metacognitive beliefs about worry. Each CAS-1R item is scored on a scale from 0 to 100 with higher scores indicating more use of unhelpful metacognitive strategies or greater conviction in metacognitive beliefs.

#### Sociodemographic and clinical data questionnaire

This questionnaire is designed to collect sociodemographic information and clinical data. Sociodemographic information will be collected only at baseline and includes age, sex, ethnicity, marital status, living arrangements, employment status and educational attainment. Clinical data to be collected at each time-point includes height, weight, smoking status, alcohol use, cardiovascular events, other health conditions, and details about medications for anxiety, depression and psychological therapies.

In addition to the measures included above, CR staff will complete a CR register for each participant detailing the number of exercise sessions and educational talks attended. The CR register will provide details to assess adherence to usual CR. Adherence to CR has been defined as the attendance to four or more sessions to each component of usual CR (exercise sessions and educational talks).

### Qualitative evaluation

Participants allocated to the intervention group will be interviewed, including those who complete the intervention, those who only complete part of it, and those who do not engage with any part of the intervention. Participants will be sampled purposively to include ranges of age and psychological distress, and levels of participation. Sampling will stop when theoretical saturation is reached. In semi-structured interviews, participants will be prompted to talk about (1) their emotional experience since the index event, (2) their reactions and expectations on being offered the intervention and (3) their experience of undertaking it.

Interviews will be semi-structured and conversational in style, and an interview guide will be used. Interviews will be conducted by telephone or face-to-face, as preferred by each participant, audio-recorded, transcribed verbatim and anonymised for analysis.

### Sample size calculation

The sample size is based on having sufficient numbers of participants to evaluate acceptability and feasibility, and to obtain provisional evidence for effectiveness. To these ends, the aim is to recruit 108 participants in total. Anticipating a 25% attrition rate, this will provide a final *n* = 40 in each arm, i.e. a total of 80 participants. With this sample, overall recruitment and retention rates for a full-scale trial will be estimated with an error of plus/minus 8% at most. This sample is also more than adequate for estimation of variability in outcome measures for which samples of 40 are generally sufficient [60].

Based on our experience of previous phases of the PATHWAY programme, 10.5 patients have been recruited for every 100 referrals, over 12 months, across three different sites. Following this figure and based on the number of annual referrals provided by the two participating sites on this phase of the PATHWAY programme, we estimate recruiting 8–9 patients per month, across both sites, over 12 months.

Feasibility trials are not powered to provide a definitive effectiveness analysis [59]. Therefore, evaluation of effectiveness will focus on effect size estimates and confidence intervals rather than statistical significance.

### Analyses

The trial will be analysed using quantitative, qualitative and economic methods.

### Quantitative analysis

To assess acceptability of Home-MCT, descriptive statistics will be used to evaluate participant ratings on the credibility, adherence and user-friendliness questionnaires, along with rates of completion of the first four modules of the Home-MCT manual. The ability to recruit and retain participants will be indicated by monthly rates of eligible patients consenting to participate and arm-specific attrition rates at 4- and 12-months follow-up.

An estimate of treatment effect size will be derived by comparing the control and intervention groups on metacognitive beliefs (MCQ-30 Positive Beliefs and Negative Beliefs subscales), CAS-1R and HADS total score at 4- and 12-months follow up, controlling for respective baseline and other relevant covariates. The analyses will use intention-to-treat principles and complete cases only, under a multivariable linear regression model.

Analysis of variability and performance of the outcome measures, coupled with recruitment and attrition rates, will be used to estimate the sample size for a full-scale RCT, including the number of sites and participants required.

All analyses will be overseen and directed by the trial statistician (DR), who will remain blind to group allocation throughout.

### Qualitative analyses

Analysis will draw on a pluralist qualitative approach. Data will be considered descriptively at first, informed by grounded theory principles [61]. We will take a more interpretative approach as analysis proceeds, going beyond line-by-line analysis to examine data in the context of the entire interview, the participant’s clinical context and the emerging analysis, and attending both to what is present and noticeably absent. Analysis will draw on constant comparison, as we iterate between the developing analysis and new interviews, and revisit earlier interviews in light of new analytic turns.

Analysis will be developed and tested by regular discussion among a core analytic team comprising a sociologist (RM) and clinical psychologists with expertise in qualitative methods (PS) and MCT (PF), and referred periodically to the broader study team. The core team will read all transcripts, and the broader team will read selected data. The framework of MCT will not be used to shape the analysis, but we will relate the final analysis to the theory of MCT that underlies the intervention. The main product of the analysis will be an understanding of contextual and intervention-related factors that promote or impair take-up of the intervention or that influence its benefits.

### Economic analysis

The economic analysis will explore the potential of Home-MCT to be cost-effective to provide a preliminary estimate of cost-effectiveness and inform the design of future evaluations. A cost-effectiveness acceptability analysis will be conducted from the perspectives of health and social care providers and patients, the key stakeholders in treatment decisions. The time horizon for the primary economic analysis will be at 12-month follow-up.

The primary measure of health benefit is Quality Adjusted Life Years, which will be estimated from the EQ-5D-5 L [56, 57] and the utility tariffs recommended by NICE at the time of the analysis [58]. At the end of the trial, a data request will be submitted to NHS Digital to obtain data on hospital-based service use covering all assessment points (baseline, 4- and 12-month follow-up). To supplement these data, the EPQ will be used at baseline, 4- and 12-month follow-up. Each item of resource use will be multiplied by the unit cost specific to that item. Standard national unit costs will be used. Single imputation will be used to impute missing baseline values for costs, utility and key covariates. Multiple imputation will be used to impute missing observations from participants who complete follow-up, and missing follow-up data for participants lost to follow-up.

The primary and sensitivity economic analyses will control for key baseline covariates or characteristics identified in the study. Cost-effectiveness acceptability curves will assess the likely cost effectiveness of the intervention and uncertainty in the observed data. This approach re-values outcomes in monetary terms. However, in the UK, there is no universally agreed monetary value for the types of outcome measures used in cost effectiveness analyses. An approach used in healthcare is to ask the question: what is the maximum amount decision-makers are willing to pay to gain one unit of outcome? An analysis of decisions made by NICE suggests a range of implicit values between £15,000 and £30,000 for the amount a decision-maker is prepared to pay to gain one Quality Adjusted Life Year. This hypothetical threshold willingness-to-pay values will be used to estimate the probability that an intervention is cost-effective.

Sensitivity analysis will assess uncertainty due to design decisions. Within trial analyses include using a 4-month time horizon, varying the assumptions and components used to estimate the unit cost of Home-MCT and using alternative measures of health benefit (primary and key secondary clinical outcomes). In addition, decision modelling will be used to extrapolate over longer time horizons of 2 and 5 years.

### Trial management and oversight arrangements

This trial is part of a larger research programme funded by the National Institute of Health Research under its Programme Grants for Applied Research scheme (RP-PG-1211 20,011). The programme is overseen by an independent programme steering committee which meets every 6 months to provide expert advice, supervise the overall programme on behalf of the National Institute for Health Research and the sponsor, and monitor progress against agreed milestones. A programme executive committee comprising the chief investigator, co-investigators, the core project team and other relevant parties meets quarterly. Notwithstanding the legal obligations of the sponsor and chief investigator, the executive committee has operational responsibility for the conduct of the trial, including monitoring overall progress to ensure adherence to the protocol and for taking appropriate action to safeguard participants and the quality of the trial. A programme management group comprising the chief investigator and core project team meet weekly to oversee the day-to-day management of the programme. There is also a service user advisory group which meets at least every 6 months and provides advice and feedback on a range of trial-related activities, e.g. reviewing study documents.

### Data management

Participants will be randomly allocated a study identity code number for use on all study documents. The research team will create a confidential database of each participant’s name, date of birth and study identity code to permit identification of participants enrolled in the study, e.g. for follow-up. All study documents will be held in strictest confidence and access to study documents will be restricted to authorised persons. Participant consent forms will be filed in the corresponding site file and in participants’ medical notes. Baseline and follow-up data, which is anonymous data, will be stored in locked filing cabinets at GMMH. These data will be entered into an electronic database for analysis purposes by study team members blind to trial arm allocation. All computers are password protected and adhere to the secure storage policies of the NHS trust and University of Manchester.

Ten percent of the data entered electronically from each of the three time points (baseline, 4- and 12-months follow-up) will be selected at random and quality checks will be performed by the statistical team. Any discrepancies will be noted, corrected and counted to obtain an error rate. Depending on the error rate, further checks will be performed.

### Safety reporting

Plans and procedures are in place for negative changes in participants’ psychological state. Health professionals delivering the Home-MCT scripted telephone calls will monitor any potential adverse events (AEs) or serious adverse events (SAEs) over the time of the intervention. Two hypothesised AEs that might occur due to the Home-MCT intervention are lowered mood and suicidality as a result of discussing current health status, reducing or terminating usual treatment in the event that a patient finds Home-MCT particularly helpful. Although it is acknowledged that thinking or talking about distress can worsen mood, this is rare and usually transient. Any AEs will be recorded at the study site using an AE form, which will be completed by the health professionals. These will be reported to the research team and reviewed for seriousness and causality by a designated sub-investigator who is not blind to treatment allocation. Any that are deemed SAEs, and are related to the intervention, will be reported to the ethics committee, the programme steering committee and the sponsor’s Research and Innovation Manager within 7 days of the event. AEs and SAEs will be reviewed on a quarterly basis at the programme’s executive committee meetings.

### Dissemination and publication policy

The main study results will be published in peer-reviewed journals and these will be made freely available online wherever possible. All presentations and publications relating to the study must be authorised by the Chief Investigator and the sponsor. Authorship of any secondary publications resulting from the study will reflect the intellectual and time input into these studies. No investigator may present or attempt to publish data relating to the PATHWAY study without prior permission from the Chief Investigator and the sponsor. The findings will also be presented at national, international and regional conferences and in public involvement events where the information from this study is relevant.

## Discussion

Forty-five percent of patients referred to CR report clinically significant levels of anxiety or depression, and most patients continue to experience these symptoms after completing CR programmes [28]. Symptoms of anxiety and depression have been associated with reduced adherence to CR programmes, reduced quality of life, increased risk of further cardiovascular events, greater health services costs, and an increased risk of mortality [3, 7–12, 14, 15]. It has been demonstrated that MCT is highly effective at reducing symptoms of anxiety and depression in mental health settings [42]. The integration of MCT delivered in a home-based self-help format into the CR programme has the potential to improve psychological, physical and economic outcomes for patients. The present study will provide data on the acceptability of the Home-MCT self-help intervention plus feasibility trial data to inform the design and sample-size for a full-scale trial. In addition, qualitative data will be obtained to understand the barriers and enablers to Home-MCT, and participants’ experiences of the intervention. Provisional estimates will also be made of the treatment effect size associated with Home-MCT, and whether it is potentially a cost-effective intervention, together with appropriate measures of uncertainty reflecting the limited ability of the study to measure these accurately.

### Trial status

The first participant was randomised to the PATHWAY Home-MCT trial on April 20, 2017. Recruitment is predicted to continue until April 2018.

## Additional file

Additional file 1: Standard Protocol Items: Recommendations for Intervention Trials (SPIRIT) Checklist. (DOC 120 kb)

## Abbreviations

AE: adverse event
CAS: cognitive attentional syndrome
CAS-1R: Cognitive Attentional Syndrome Scale-1 revised
CR: cardiac rehabilitation
EPQ: Economic Patient Questionnaire
GMMH: Greater Manchester Mental Health NHS Foundation Trust
HADS: Hospital Anxiety and Depression Scale
Home-MCT: Home-Metacognitive Therapy
IES-R: Impact of Event Scale-Revised
MCQ-30: Metacognitions Questionnaire 30
MCT: metacognitive therapy
NHS: National Health Service
SAE: serious adverse event
